# 

**Published:** 1816-12

**Authors:** 


### CORRESPONDENCE.

Dr. Palmer's Cases of Croup; Dr. Kennedy's Description of an Intestinal Concretion; and Mr. Ward's Case of Urinary Calculus, complicated with a large abdominal Tumour, are postponed for want of room. Dr. Scudamore's work is received, and will be reviewed in our February Number.

We are extremely obliged to Mr. Coley, of Cheltenham, for his valuable communication. This most interesting document, authenticated by the testimony of Lord Exmouth himself, probably, will be found to transfer the honour of *first tying the Carotid Artery* from Mr. Abernethy to the late Mr. Fleming, Surgeon in the Royal Navy. Independently of this, it is perhaps one of the most interesting cases ever laid before the medical public. It shall have an early insertion.

HÆMALOGIA; or, Practical Observations on the different States of the Circulation in Health and in Disease, in a Series of Essays, will appear in the ensuing Volume.

We return thanks to Dr. Quarrier for the drawing of Borghini's head, preserved in the University of Marseilles, and mentioned in the article "*Cas Rares,*" *New Medical and Physical Journal,* vol. x. We shall probably take an opportunity of presenting our readers with an outline of it.

The Title and Index to this Volume will be given in our next Number.

☞ Authors and Publishers are requested to send Copies of new Works for Review as early as possible after their publication.

*The Medico-Chirurgical Journal has closed its first year's labours. The Editors can contemplate the past with pleasure; the future with confidence. They fearlessly assert, that the two first Volumes of their Journal present a mass of information, a style of composition, an order of arrangement, and a choice of materials, unequalled by any work of similar extent. The increasing reputation of the Medico-Chirurgical Journal demonstrates the public appreciation of its*

*Editorial labours. These will not be relaxed, but increased. They have the pleasure of announcing, that the* Original Department *will, in future, assume an attitude of importance and respectability not inferior to that of any contemporary Journal.*

### INDEX.

A

ABDOMINAL disease, singular case of 3
Abortion, observations on the various means of producing 420
——— on the signs of 422
——— provoked, on the legislation concerning 497
——— case of 505
——— pathology of ib.
Acid, sulphuric, and indigo, case of poisoning by 157
Alkalies, poisonous property of 41
Ammonia, case of poisoning by 154
Anatomy, morbid, abridgement of Morgagni's 159, 254, 430, 513
Aneurism of the aorta, cases of, 94, 210,
——— popliteal, case of 462
Aorta, cases of aneurism of the, 94, 210
Apoplexy, sanguineous, cases of 163, 255
——— serous, cases of 430
Ardent spirits, the effects of, on the stomach 213
Arrowsmith, Mr. on the irritable bladder 11
Arsenic, on the means of detecting 171
Arterial pulse, on the nature and cause of 22
Arteries, mode of tying in operations 28
——— observations on the ligature of 234
——— peculiar mode of stopping hæmorrhage from the 263
Ascites, treated by mercurial friction 215

B

*Badé Sumoom*, description of 350
Bailey's, Mr. case of anomalous disease 177
——— fatal case of diarrhœa 278
——— case of enlarged liver 452
Baillie, Dr. on spasmodic stricture of the sphincter ani 132
——— observations on the green jaundice 133
——— on a particular species of purging 134
——— Mr. H. on hemeralopia 179
Bandage, new, for transverse wounds of the throat 83
Bandages, observations on 395
Baynard's, Dr. poem on health 65
Bedingfield's, Mr. compendium of medical practice 100, 207, 304
Bird's, Mr. case of uterine polypus 455
Black drop, original recipe for 352
Bladder, irritable, observations on 11
——— cases of 13
——— various affections of 284
——— treatment of 287
Blane, Sir Gilbert, on the comparative health of the Brish navy 226
Bleeding, utility of, in hæmoptysis 109
——— after leeches, easy method of stopping 262
Blood, on the circulation of 355
——— process for obtaining the colouring principle of 509
Blumenbach's, Dr. letter read at the Royal Society of Gottingen 79
Bodies, medico legal examination of 68
Bones, fractured, in a new-born infant 262
BOOKS, NEW, ANNOUNCED.—*Journal Universel des Sciences Medicales*, 175. Dr. Adams's Account of Mr. Hunter's Life, Doctrine and Opinions, 263. Mr. Accum's Practical Essay on Chemical Re-agents, 263. Oracular Communications, by Æsculapius, 264. Mr. Howship's Practical Observations on Surgery and Morbid Anatomy, 440. Mr. Pettigrew's Memoirs of Dr. Lettsom, ib.
BOOKS REVIEWED.—Dr. Parry's Experimental Inquiry into the Nature, Cause, and Varieties of the Arterial Pulse, 18. Medico-Chirurgical Transactions, vol. VI. 28. M. Orfila's System of Toxicology, 40. M. Roux's *Relation d'un Voyage fait à Londres en* 1814, 53, 290. Dr. Baynard's Poem on Health, 65. Mr. Bedingfield's Compendium of Medical Practice, 100, 207, 304. Mr. Doughty's Observations and Enquiries into the Nature and Treatment of Yellow or

Bulam Fever, 115. Medical Transactions of the College of Physicians, 123, 314. Medico-Chirurgical Transactions, vol. vi. 137, 226. Mr. Waller's Treatise on Incubus or Nightmare, 188. Mr. Whateley's Practical Treatise on Wounds and Ulcers of the Legs, 198. Dr. Reid's Essays on Nervous Affections, 218, 386. Dr. Burnett's Practical Account of the Mediteranean Fever, 235. Mr. Howship on the Diseases of the Urinary Organs, 280, 396. Mr. Ring's Answer to Dr. Kinglake, 335. Dr. Lambe's Additional Reports on the Effects of Regimen, 371, 466. Mr. Jardine on the Improvement of Surgical Instruments, 392. Dr. Henning's Critical Enquiry into the Pathology of Scrofula, 405. Dr. Hutchison's Observations in Surgery, 458. Mr. Norman's Reply to Dr. Kinglake, 478. Dr. J. R. Johnson on the Medicinal Leech, 481.

Brain, case of diseased 271
——— cases illustrating the pathology of the 314, 347
Brenan, Dr. on Puerperal Fever 450
Brodie, Mr. on ulceration of the cartilages of the joints 137
Bronchitis, case of, and dissection 109
Bronchotomy, cases of operation of 33
Bulam fever, description of 238
Burnett's, Dr. Practical Account of the Mediterranean fever 235

C

Cachexia aphthosa, remarks on 129
Calculus, large, removed from the female urethra 229, 289
Cambridge, account of the fever prevailing at 326
Cantharides, poisonous properties of 45
——— chemical analysis of 87, 171
Carditis, on the symptoms and treatment of 113
Cartilages of the joints, on the ulceration of 137
Cellular membrane, induration of 70
Cerussa successfully applied in tic douloureux 105
Cholic, on the Indian method of treating 526
Chorea cured by oxyd of zinc 105
——— case of 137
Chyle, experiments on the chemical nature of 233
Cinchona, French substitute for 84
Circulation of the blood illustrated 359
Clarke, Dr. on certain articles of food
Cloven-footed children, method of curing 527
Cold water, on the use of, in gout 478
Colica pictonum cured by nitrate of silver 127
Colouring principle of the blood, process for obtaining 509
Compendium of medical practice 100, 207, 304
Contagious nature of yellow fever, discussion concerning the 517
Convulsive affection, cases of 325
Correspondence 88, 175, 265, 354, 441, 529
Cow-pox, effects of, in mitigating the malignity of small-pox 327, 440
Cranial bones, separation of 270
Croup, case of, violent 439
Cynanche parotidea contagious in some cases 182

D

Death, on the pathognomonic signs of 334
Denmark's, Dr. observations on the Mediterranean fever 39
Dewar's, Mr. case of organic disease of the stomach 89
Diabetes, case of 124, 304
Diarrhœa, fatal case of 278
Dictionary, French, extracts from 70, 340, 420, 497
Digitalis of doubtful utility in hæmoptysis 110
Disease, anomalous, case of 177
Doughty, Mr. on the Bulam or yellow fever 115
Dysentery, division of into species 141

E

Electric phænomenon observed in Spain 82
Elephantiasis, account of cases of 32, 322
*Endurcissement du tissu cellulaire*, description of 70
——— cases of 71
Epidemic at Cadiz, case of 121
Epilepsy, on the various remedies of 128
——— remarks on 318
Erlangen Physico-Medical Society, diplomas of 173
Erysipelas, fatal, under the Brunonian treatment [illegible]12
Experiments to elucidate the cause of the pulse 20
Eye, case of painful affection of 526

F

Falx of the dura mater ossified 258
Fermented liquors, on the use of 381
Fever, Mediterranean, observations on 39
——— yellow or Bulam, observations on 115, 443, 517

Fever in the Peninsula, remarks on 141
——, Mediterranean, practical account of 235
—— on the theory of 309
—— puerperal, cases of 323, 450
—— attended with extraordinary appetite 324
—— Cambridge, cases of 326
—— puerperal, cured by spirit of turpentine 450
Fistula in ano, mode of operating for, in France 299
Food, comparison between vegetable and animal 376
Foy's, Mr. case of melancholia 267
Fractures, French method of treating 294
Fungus hæmatodes in the bladder, case of 289

G

Galvanism, recommended in disorders of the eyes 350
Gastritis, case of, and dissection 213
Glass powdered, poisonous properties of 45
Gottingen, proceedings at the Royal Society of 79
Gout succeeding to renal inflammation 283
—— misplaced, fatal case of 284
Graham's, Mr. case of mortification of the uterus 231
Gratiola, recent, injurious effects of 83
Gumprecht, Dr. on the *Lactuca virosa* 232

H

Hæmoptysis, on the treatment of 109
Hæmorrhage, secondary, case of 461
Harrison's, Dr. cases of Cambridge fever 326
Haviland's, Professor, observations on the Cambridge fever 326
Head, case of water in the 135
—— birth of the, destitute of a body 167
Head-ach, fatal cases of 160
Health, a poem on 65
Heart, on the inequality of action of 209
—— case of malformation of the 439
Heberden's Dr. contrivance for bed-ridden people 127
—————— case of water in the head 135
Hemeralopia, observations on 179
Hemiplegia, on the treatment of 104
*Hernia ventriculi*, case of 140
Henning, Dr. on scrophula 405
History, medical, of the British army in the Peninsula 140
Hooping-cough cured by the *lactuca virosa* 232
Hospitals, London, remarks on the practice of 58
Howship's, Mr. case of aneurism of the aorta 94
—————— on the diseases of the urinary organs 280, 396
Hutchison's, Dr. observations in surgery 458
Hydrocele, extraordinary cases of 78
Hydrocephalus internus, case of 102
Hydrogenized sulphuret of potash, remarks on 51
*Hydrops pericardii*, case of 208
Hydro-renal distension, case of 3
Hydrothorax, on the various modes of treating 112

I & J

Incubus, fatal case of 190
Indigo and sulphuric acid, case of poisoning by 157
Inflammation of the pia mater, case of 104
Instrument for extracting fish bones, &c. 394
Interment, premature, among the Moors 351
Intermittents, on the Indian method of treating 525
Iodine, description and habitudes of 51
Irritation of the bladder from stricture in the rectum 288
Jacksonian prize of the College of Surgeons 174
Jardine, Mr. on surgical instruments 392
Jaundice, green, observations on 133
Johnson's, Mr. case of abdominal disease 3
—————— Dr. on the circulation of the blood 359
—————— Dr. J. R. on the medicinal leech 48[illegible]
Joints, on the ulceration of the cartilages of 137
Journals, foreign, extracts from 68, 154, 347, 505
Jurisprudence, medical, observations on 68, 146, 340, 497

K

Kidney, enormously enlarged 7
—— different affections of, and their treatment 282
—— on the functions of the 305

L

*Lactuca virosa* useful in hooping-cough 232
Lambe, Dr. on scrofula and consumption 371, 466
Larynx, on some affections of 33
—— case of disease of 437
Latham, Dr. on leucorrhœa 126
—————— on epilepsy and worm medicines 128

Latham, Dr. on cachexia aphthosa 129
——— on the superacetate of lead 324
Lawrence, Mr. on the ligature of arteries 28
——— on affections of the larynx 33
Lead, poisonous properties of 47
——— on the use of the superacetate of 324
LECTURES ANNOUNCED.—Dr. Clough, on Midwifery, 88, 352. Drs. P. M. Latham and Southey, on the Practice of Physic, 175. At St. Thomas's and Guy's Hospitals, 264. At St. Bartholomew's Hospital, ib. At St. George's Medical School, ib. Dr. Adams, on Medicine, 265. Mr. Carpue, on Anatomy, ib. Dr. Clutterbuck, on Medicine, ib. Mr. Wilson and Mr. Bell, on Anatomy and Surgery, ib. Mr. Taunton, on Anatomy and Surgery, ib. Mr. Guthrie, on Surgery, 352. Drs. Merriman and Ley, on Midwifery, 353, 529
Lee's Dr. case of puerperal fever 323
Leech, natural history of 485
——— anatomical structure of 490
——— diseases, preservation, and management of 493
Lenticular, improved one 393
Leucorrhœa, observations on 126
Ligature of arteries, observations on 234, 463
Liver, enormously enlarged 452
*Lumbrici*, case of 216

M

M'Grigor's, Sir James, medical history of the British armies in the peninsula 140
Mania, pathology of 341
Marcet's, Dr. experiments on chyle 233
Maton's, Dr. description of a rash mistaken for scarlatina 134
——— case of chorea 137
Medical Journal, new French one 88
——— topography of New Orleans, remarks on 96
——— practice, compendium of, 100, 207, 304
——— Transactions of the College of Physicians 123, 314
——— jurisprudence, observations on 68, 164, 340, 497
Medico-Chirurgical Transactions, review of 28, 137
Medico-legal examination of bodies, 68, 146
Mediterranean fever, observations on 39
Melancholia from a material cause in the brain 267
Membrane, cellular, induration of 70
Membranes-pseudo, formation of 528
Meteorological Register for Harwich 176, 264, 353, 442, 530
Millington's, Mr. case of natural small-pox 137
*Mirage*, description of 351
Miscellanies, Medical, 81, 159, 254, 349, 430, 513
Moira, saline water, chemical analysis of 352
Morbid Anatomy, abridgement of Morgagni's work on 159, 254, 430, 512
Mortimer, Mr. letter from, on the Bulam fever 443
Moxa, beneficial application of 56
Muriatic acid, a cure for calculous phosphates 284
Musk, case of chorea cured by 137

N

New Orleans, on the medical topography of 96
Night-mare, description of 191
——— proximate cause of 194
Nitrate of silver useful in colica pictonum 127

O

Obituary 88, 175, 265, 354, 441, 521
Observations on an organic disease of the stomach 92
——— by the Editors, on Dr. Palloni's case 186
Opium, on the habitual use of 223
——— new preparation of 263
*Orban*, M. on phthisis 321
Orfila, M. on poisons 40
Orleans, New, on the medical topography of 96
Oysters, the injurious effects of in child-bed 132

P

Palloni's, Dr. case and dissection 183
Palmer's, Dr. case of diseased brain 271
Palpitations, remarks on 318
Parallel between French and English surgery 54, 290
Paralysis, remarkable case of 386
Paralytic affections, remarks upon some cases of 131
Parry, Dr. on the arterial pulse 18
——— on the circulation of the blood 355
Peritonitis, remarks upon 215
Phænomenon, singular, on Mount Ætna 81
Phrenitis cured by bleeding 104
Phthisis, on the effect of climate in 144
——— extracts from a paper on 321

### INDEX.

Physicians, Medical Transactions of the College of 123
Pia mater, case of inflammation of 104
Pneumonia, on the practice of bleeding in 143
Poisoning by oil of vitriol, case of 247
Poisons, description of various 41
——— astringent, enumeration of 47
Powell, Dr. on paralytic affections 131
——— on the pathology of the brain 314
———'s, Dr. cases of convulsive affection 325
Polydipsia, case of 252
Polypus, uterine, case of 455
Pregnancy, on some affections resembling 84, 261
Prostate gland, on the diseases of 396
Pseudo membranes, on the formation of 528
Puerperal fever, case of 323
——— cured by spirits of turpentine 450
Pulmonary artery, on the functions of 211
——— consumption, on the treatment of 333
Pulse, inquiry into the nature and causes of 18
——— cessation of at both wrists 349
Purging, on a particular species of 134
Purpura hæmorrhagica, case of 332

R

Raspatory, method of improving 392
Rectum, on the affections of 306
Reid, Dr. on hypocbondriacal and nervous affections 218
Register, Meteorological, for Harwich 176, 264, 353, 442, 530
Remarks upon an affection resembling pregnancy 84
Remorse not always the effect of guilt 222
Rheumatism, chronic, cured by ol. terebinth. 145
Rindler's, Dr. method of curing cloven-footed children 527
Ring's, Mr. answer to Dr. Kinglake, on the cooling treatment of gout 335
Roberts, Dr. on colica pictonum 127
——— case of elephantiasis 322
——— observations on consumption 333
Romer, Dr. on the pathognomonic signs of death 334
Roux's, M. parallel of French and English surgery 59, 290
*Rubeola*, treatment of 311

S

Satterly's, Dr. cases of diabetes 124
——— case of fever attended with extraordinary appetite 324
Scarlatina, sometimes confounded with a different rash 134
——— fatal case of and dissection 301
Scrofula, inquiry into the pathology of 405
——— surgical treatment of 414
——— medical ditto 416
Selections 68, 146, 247, 340, 420, 497
Shoveller's, Mr. cases of fever 368
Simulated pregnancy, case of 261
Small-pox, recurrence of 137
Society for relief of widows and orphans, meeting of 440
Somnambulism, case of 333
Spasmodic stricture of the urethra 399
Sphacelation consequent on uterine polypus 455
*Sphincter ani*, on the spasmodic stricture of 132
Spine, on the diseases of 308
——— case of fracture of ib.
Spleen, cartilaginous texture of 178
Statements of the comparative health of the British navy 226
Statistics, medical, of Paris 168
Stevenson's, Mr. case of affection of the eye 526
Stomach, case of organic disease of 89
——— case of ulceration of 212
——— the effects of ardent spirits on 213
Sulphuric acid, case of poisoning by 247
Surgery, parallel between French and English 59

T

Teeth, improved instrument for extracting 393
Tetanus cured by exposure to extreme cold 145
———, case of 334
Tic Douloureux cured by the application of cerussa 105
Tinea capitis, remedies for 101
Tongue, on the irritability of 80
Tooth-ach, remedy for 107
Transverse wounds of the throat, new bandage for 83
Trephine, improvement of suggested 392
Travers, Mr. on the ligature of arteries 234
Tumour, abdominal, remarkable case of 270
Turpentine, spirit of, employed in puerperal fever 450
Tympanites, mercurial friction useful in 216

U

*Ulceratio pharyngis*, case of and dissection 107
——— *laryngis*, case of and dissection 108
Ulceration of the cartilages, observations on 137
Ulcers of the legs, practical observations on 198
Urethra, worms discharged from alive 249
Urinary organs, on the diseases of 280
Uterine polypus, case of 455
Uterus, case of mortification of the 231
——— on the affections of the 307

V

Vaccine virus, on the best mode of preserving 173
Vaughan's, Dr. case of tetanus 334
Ventricles of the heart, inflammation of the 208
——— of the brain, case of effusion in the 271
*Ventriculi hernia*, case of 140
Verdigris, case of poisoning by 82
Vertebræ, case of dislocation of the 77
Villermi, Dr. on the formation of pseudo-membranes 528
Vision, quadruple, case of 262
Von Embden's, Dr. case of abdominal tumour 270

W

Walker, Dr. on preserving vaccine virus 173
Waller, Mr. on Incubus or night-mare 188
Water in the head, case of 135
Whately, Mr. on wounds and ulcers of the legs 198
Wheelwright's, Mr. case of *hernia ventriculi* 140
Worm medicines, remarks upon 128
Worms contained in the urinary passages 249
Wounds, transverse of the throat, account of new bandage for 83
——— of the legs, practical observations on 198

Y

Yeats's, Dr. case of *purpura hæmorrhagica* 332
——— case of somnambulism 333
Yelloly's, Dr. case of calculus 229
Yellow fever, observations on 115, 517
Young, Dr. on palpitations and on epilepsy 318

Z

Zinc, oxyd of, useful in chorea 105

### REFERENCE to the PLATE.

Dr. Johnson's Apparatus, illustrating the Circulation of the Blood, page 359.

PRINTED BY W. THORNE, RED LION COURT, FLEET STREET.

### ORNAMENTED LABELS,

FOR

### Surgeons, Apothecaries, and Druggists,

PRINTED AND SOLD BY

### E. COX AND SON,

ST. THOMAS's-STREET, BOROUGH.

COMPLETE sets of ORNAMENTED LABELS, printed on coloured and white paper, were published previous to the appearance of the London Pharmacopœia of 1809, containing all the Names of Medical Preparations then in use; these Labels have been from the first very well received, and very extensively employed, both in Town and Country. On the appéarance of the above Pharmacopœia, however, it became necessary to print a new Set, containing the Names of all those Articles which had been changed by the College; this was immediately done, and in the same style of execution and elegance as the former ones. This Set, containing only the Names changed, and those newly introduced, was sold separately.

An inconvenience however, arising from this circumstance, to those gentlemen who were not in possession of the former set, the old names of which were to many superfluous, the Publishers have just formed *Complete New Sets,* adapted to the last Edition of the London Pharmacopœia of 1815.

These Labels are printed in three different sizes, which may be had either plain or coloured. The first, or largest size, is calculated for Druggists, and large Dispensaries; the second answers extremely well for the generality of Retail Shops, and the third or smaller size, forms an elegant and convenient collection of Labels for Surgeons, Apothecaries, &c. who have small private shops.

These Labels will be found much cheaper than paint; and if any of them should be at any time lost or destroyed, single Labels of any description may be had, by applying to the publishers. These Labels may be made to last many years, if covered with varnish, and their beauty will, by that means, be greatly increased.

| | £. | s. | d. |
|---|---|---|---|
| Complete Set, in Three Sizes, White Paper | 1 | 16 | 0 |
| Ditto, French Green Paper | 2 | 10 | 0 |
| Ditto, Patent Yellow Paper | 2 | 10 | 0 |
| Set of Labels, First Size White Paper | 0 | 18 | 0 |
| Ditto, French Green Paper | 1 | 4 | 0 |
| Ditto, Patent Yellow Paper | 1 | 4 | 0 |
| Set of Labels, Second Size, White Paper | 0 | 14 | 0 |
| Ditto, on French Green Paper | 0 | 18 | 0 |
| Ditto, Patent Yellow Paper | 0 | 18 | 0 |
| Set of Labels, Third Size, White Paper | 0 | 10 | 0 |
| Ditto, French Green Paper | 0 | 14 | 0 |
| Ditto, Patent Yellow Paper | 0 | 14 | 0 |

*Lately Published,*

Books of Labels of the Edinburgh Names according to the last Pharmacopœia.

Set of Labels, white paper - 12s

——————— green paper - 18s

——————— Yellow paper - 18s

These are of the same pattern as those of the London Pharmacopœia.

### LIST OF LABELS

### *Contained in the Book.*

ABIET. RESIN.

Acaciæ Gum.

Acet. Colch.

Acet. Scill.

Acid. Acetic.

Acid. Benz.

Acid. Citric.

Acid. Mur.

Acid. Nitric.

Acid. Nit. Dil.

Acid. Sulph.

Acid Sulph. D.

Adeps Præp.

Alcohol.

Aloes. Hepat.

Alumen.

Alum. Exsic.

Alum. Pur.

Alum. Rup.

Ammon. Carb.

Ammon. G. R.

Ammon. Mur.

Ammon. Subcarb.

Amygd. Amar.

Amygd. Dul.

Amylum.

Antim. Crud.

Antim. Calc.

Antim. Mur.

Antim. Oxyd.

Antim. Sulp.

Ant. Sulph. Præc.

Antim. Tart.

Argent. Nit.

Arnatto.

Arsenic. Alb.

Arsen. Ox. Sub.

Assaf. Gum. Res.

Aq. Alex. Sp.

Aq. Anethi.

Aq. Cinnam.

Aq. Carui.

Aq. Distill.

Aq. Flor. Aur.

Aq. Flor. Sam.

Aq. Fœnic.

Aq. Fontan.

Aq. Hungar.

Aq. Hyssopi.

Aq. Lavand.

Aq. Menth. Pip.

Aq. Menth. Vir.

Aq. Mellis.

Aq. Pimentæ.

Aq. Pulegii.

Aq. Rosæ.

Aq. Theriac.

Aq. Zinc. Vc C.

Æther. Rect.

Æther. Sulph.

Ærugo. Aris.

Ærugo. Distil.

Ærugo. Pulv.

Bacc. Junip.

Bacc. Lauri.

Bals. Anod.

Bals. Guaiac.

Bals. Locat.

Bals. Peruv.

Bals. Styrac.

Bals. Tolu.

Benzoinum.

Bol. Armen.

Bol. Cardiac.

Bol. Diuret.

C. Cinch. Cord.

C. Cinch. Lanc.

C. Cinch. Oblo.

C. Cuspariæ.

Calam. Ppt.

Calcis. Mur.

Calx.

Cambogia.

Camphora.

Can. Chel. Pp.

Can. ocul. Pp.

Cap. Pap. Alb.

Capsic. Baccæ.

Carbo. Ligni.

Caryophyl.

Cera. Alb.

Cerat. Calam.

Cerat. Cetac.

Cera. Flav.

Cerat. Lyttæ.

Cerat. Plumb. C.

Cer. Plumb Sub.

Cerat. Resinæ.

Cerat. Sabin.

Cerat. Sapon.

Ceratum.

Cetaceum.

Chel. Canc. C.

Coccus.

Cocul. Ind.

Colocynth.

Conf. Amygd.

Conf. Aurant.

Conf. Arom.

Conf. Cassiæ.

Conf. Damoc.

Conf. Opii.

Conf. Rosæ. Can.

Conf. Rosæ. Gal.

Conf. Rutæ.

Conf. Scamm.

Conf. Sennæ.

Conii Folia.

Copaiba.

Copal. Res.

Corall. Ppt.

Coral. Rub.

Cort. Aur.

Cort. Cascar.

Cort Canellæ.

Cort. Cinch F.

Cort. Cinnam.

Cort Cinch. R.

Cort. Cinch.

Cort. Granat.

Cort. Limon.

Cort. Querc.

Cort. Sassaf.

Cort. Ulmi.

Cornu. Ustum.

Corks.

Creta. Ppt.

Croc. Antim.

Crocus.

Cubebæ.

Cuprum. Amm.

Cupri. Sulph.

Dec. Aloes. C.

Dec. Cinchon.

Dec. Dulcam.

Dec. Hordei. C.

Dec. Lichen.

Dec. Papav.

Dec. Querc.

Dec. Sarsap.

Dec. Sarsap. C.

Dec. Senegæ.

Dec. Veratr.

Dec. Ulmi.

Diapente

Dolichi. Pub.

Eau. De. Luce.

Eau De. Arq.

Elec. e, scord.

Elemi. Res.

Elix. Vit. A.

Emp. Ammon.

Emp Am. c Hyd.

Emp Ceræ.

Emp. Cumini.

Emp. Galb. C.

Emp. Hydrar.

Emp. Lyttæ.

Emp. Opii.

Emp. Picis. C.

Emp. Plumb.

Emp. Resin.

Emp. Sapon.

Emp. Thuris. C.

Emp. Lad. C.

Ess. Amberg.

Ess. Menth. Pip.

Euphorb. G. R.

Ex. Aconiti.

Ex. Aloes. P.

Ex. Aloes. Vulg.

Ex. Aloes Spic.

Ex. Anthem.

Ex. Bellad.

Ex Colocyn.

Ex. Conii.

Ex. Catechu.

Ex. Cinch. Res.

Ex. Cascar.

Ex. Colocyn. C.

Ex. Cinchon.

Ex. Elaterii.

Ex. Glycyrrh.

Ex. Gentian.

Ex. Hæmatox.

Ex Helleb. N.

Ex. Humuli.

Ex. Hyoscy.

Ex. Jalapæ.

Ex. Opii.

Ex. Papaver.

Ex. Rhei.

Ex. Rutæ.

Ex. Sabinæ.

Ex. Sarsap.

Ex. Sennæ.

Ex. Taraxac.

Ferr. Ammon.

Ferr. Limat.

Ferr. Subcarb.

Ferr. Sulph.

Ferr. Præcip.

Ferr. Rubig.

Ferr. Tart.

Fist. Armat.

Flo. Anthemid.

Flor. Samb.

Flor. Rosar. G.

Fol. Sennæ.

Fol. Belladon.

Fruct. Tamar.

G Assafœt.

G Shell. Lac.

G. Tragacan.

Gallæ.

Galban. G. R.

Glo. Gascoig.

Gran. Parad.

Guaiac. Res.

Hord. Perlat.

Hyd. Acetas.

Hyd. c. Creta.

Hyd. Mur. Mit.

Hyd. Nitr. Ox.

Hyd. Ox. Ciner.

Hyd. Ox. Rub.

Hyd. Oxymur.

Hyd Præc. Alb.

Hyd. Purif.

Hyd. Submur.

Hyd. Sulp. N.

Hyd. Sulph. R.

Hyd. Sulphas.

Ichthyocol.

Ichthyoc. In.

Ind. Arrow. Root.

Inf. Anthem.

Inf. Armor. C.

Inf. Aurant. C.

Inf. Calumb.

Inf. Caryoph.

Inf. Cascar.

Inf. Catechu. C.

Inf. Cinchon.

Inf. Cuspar.

Inf. Digital.

Inf. Gent C.

Inf. Lini.

Inf. Quass.

Inf. Rhei.

Inf. Rosæ.

Inf. Sennæ.

Juniper. Res.

Kino. G. R.

Lichen.

Lign. Hœmatox.

Lign. Quass.

Lign. Guaiac.

Lin. Amm. Fort.

Lin. Amm. Subc.

Lin. Camph. C.

Lin. Camph.

Lin. Ærug.

Lin. Hydrarg.

Lin. Sapon. C.

Lin. Tereb.

Lint. Opt.

Liq. Alum. C.

Liq. Ammon.

Liq. Ammon. A.

Liq. Ammon. C.

Liq. Ant. Tart.

Liq. Arsen.

Liq. Calcis.

Liq. Calc. Mur.

Liq. Cup. Amm.

Liq. Fer. Alk.

Liq. Hyd. Oxym.

Liq. Plumb. S. A.

Liq. Potassæ.

Liq. Pot. Subc.

Liq. Vol. C. C.

Lap. Gascoig.

Lap. Contray.

Lozenges.

Lyttæ.

Magnesia.

Magnes. Carb.

Magnes. Sulph.

Manna. Com.

Manna. Flak.

Manna. Sic.

Manna. Opt.

Mastich. Res.

Mel Ang.

Mel. Borac.

Mel. Despum.

Mel. Rosæ.

Mel. Scillæ.

Milleped. Pp.

Mist. Ammon.

Mist. Amygd.

Mist. Assaf.

Mist. Camph.

Mist Cretæ.

Mist. Ferr. C.

Mist. Guaiac.

Mist. Mosch.

Mucil Acac.

Mucil Amyli.

Myrrha.

Myrrh. G. R.

Nux. Vomic.

Ol. Amygdal.

Ol. Animal.

Ol. Anisi.

Ol. Anthem.

Ol. Æther.

Ol. Bergam.

Ol. Bac. Laur.

Ol. Cajuputi.

Ol. Carui.

Ol. Caryoph.

Ol. Cinnam.

Ol. Camphor.

Ol. Fœnic. D.

Ol. Junip. Bac.

Ol. Lavandu.

Ol. Lini. S. I.

Ol. Limon.

Ol. Menth Pip.

Ol Menth. Vir.

Ol. Myrist.

Ol. Origan.

Ol. Olivæ. Opt.

Ol. Olivæ. Com.

Ol. Palm.

Ol. Petrol.

Ol. Pulegii.

Ol. Piment.

Ol. Rhodii.

Ol. Ricini.

Ol. Roris-mar.

Ol. Rosar.

Ol. Rutæ.

Ol. Sabinæ.

Ol Sass. Rad.

Ol. Sem. Cym.

Ol. Sem. Anethi.

Ol. Sinapeos.

Ol. Spicæ.

Ol. Succin. C.

Ol. Sulph.

Ol. Sulph. A.

Ol. Tereb. R.

Ol. Viride.

Oliban. G. R.

Opium.

Opopon. G. R.

Os. Sepiæ.

Oxymel.

Oxymel. Scil.

P. Acaciæ.

P. Aloes. C.

P. Aloe c Fer.

P. Aloe c Can.

P. Amygdal. A.

P. Amygdal.

P Antimonial.

P. Arsen. Alb.

P. Asari. C.

P. Bac. Lauri.

P. Basilic.

P. Bol. Armen.

P. Calumbæ.

P. Carb. Ligni.

P. Castor. R.

P. Castor. N. A.

P. Cascaril.

P. Canel. Alb.

P e Cerus. C.

P. Cetacei.

P. Chel. Canc. C.

P. Cinnam. C.

P. Cinch. Oblo.

P. Cinch. Cord.

P. Cinch. Lanc.

P. Colocynth.

P. Contray. C.

P. Corn. Ust c O.

P. Cretæ. C.

P. Cretæ C c O.

P. Curcum.

P. Cuspariæ.

P. Diapent.

P. Digital.

P. Enula. C.

P. Fœnugric.

P. Gallæ.

P. Gentian.

P. G. Guaiac.

P. G. Myrrh.

P. Glycyrrh.

P. Helle[illegible].

P. Irid. Flor.
P. Ipecac.
P. Ipecac. C.
P. Jalapæ.
P. Kino. C.
P. Lytta.
P. Lap Hybern.
P. Lyttæ.
P. e Myrrh. C.
P. Opii.
P. Salep.
P. Sang. Dracon.
P. Scammon. C.
P. Scam c Cal.
P. Scam. C c Aloe.
P. R. Scillæ. S.
P. Sem. Cymini.
P. Sem. Lini.
P. Sem. Anisi.
P. Sem. Staph.
P. Sennæ. C.
P. Stanni.
P. Succin. C.
P. Tragac. C.
P. Tragacan.
P. Valerian. S.
P. Uva. Ursi.
P. Zingib.
Phials.
Pil. Aloes. C.
Pil. Alo. c Myrrh.
Pil Camb. C.
Pil. Colo. S.
Pil. Colo c Al.
Pil. Fer. Comp.
Pil. Galban. C.
Pil. Hydrar.
Pil. Hyd. Subm. C.
Pil. Opii.
Pil. Sapon c Op.
Pil. Scillæ. C.
Pimentæ.
Pip. Alb.
Pip. Long.
Pip. Nigrum.
Pix Arida.
Pixides.
Plumb. Oxy. Sem.
Plumb. Subc.
Plumb. Sup. Ac.
Potass. Acet.
Potass. Carb.
Potass. c Calc.
Potass. Fusa.
Potass. Imp.
Potass. Nit.
Potass Subcarb.
Potass Sulphas.
Potass Sulphuret.
Potass. Sur. Sulph.

Potass. Tart.

Pterocar. Lign.

Pulv. Conii.

Pulv. Potas. Nit.

Pulv. Rhei.

Pulv. Veratri.

R. Anchusa.

R. Angelic.

R. Althæ.

R. Bistor.

R. China.

R. Contray.

R. Curcum.

R. Enulæ. C.

R. Eryngium.

R. Galang.

R. Gentian.

R. Ginseng.

R. Glycyrrh.

R. Helleb. N.

R. Ipecac.

R. Irid. Flor.

R. Jalapæ.

R. Mezer.

R. Pyreth.

R. Rubia. Tr.

R. Sars. Inc.

R. Salep.

R. Scilla.

R. Serpent. V.

R. Torment.

R. Valer. S.

R. Zedoar.

R. Zingib.

Rad. Calami.

Rad. Calumbæ.

Rad. Rhei.

Rad. Senegæ.

Rad. Spigeliæ.

Rad. Veratri.

Ras. Corn. C.

Ras. Guaiac.

Ras. Sassaf.

Resina. Fl.

Resin. Jalap.

Resina. Nig.

Rhæad. Pet.

Sal. Acetos.

Sal. Polychrest.

Sal. Prunel.

Sandrach. G. R.

Sang. Dracon.

Sago.

Sagapen. G. R.

Sarcocol G. R.

Sapo. Castil.

Scammon. G R.

Sem. Anethi.

Sem. Anisi.

Sem. Card. Maj.

Sem. Card. Min.

Sem. Carui.

Sem. Coriand.

Sem. Cydonior.

Sem. Cymini.

Sem. Dauci.

Sem. Fœnug.

Sem. Fœnicul.

Sem. Lini.

Sem. Pæoniæ.

Sem. Santon.

Sem. Sinap.

Sem. Staph.

Senega. Res.

Sevum. Præp.

Sodæ. Carb.

Sodæ. Subbo.

Sodæ. Sulph.

Sodæ. Phosph.

Sodæ. Tart.

Sod. Subcarb.

Sod. Subcar. Ex.

Sp. Ammon. Ar.

Sp. Ammon. S.

Sp. Ammon.

Sp. Amm. Fœt.

Sp. Anisi.

Sp. Armor. C.

Sp. Æth. Nit.

Sp. Æther. A.

Sp. Æther. S.

Sp. Æther. S. C.

Sp. Bryon. C.

Sp. Camphor.

Sp. Carui.

Sp. Cinnam.

Sp. Cochlear.

Sp. Junip. C.

Sp. Lavand.

Sp. Lavand. C.

Sp. Ment. Pip.

Sp. Menth. Vir.

Sp. Myrist.

Sp. Pimentæ.

Sp. Pulegii.

Sp. Raphan. C.

Sp. Rectif.

Sp. Rorismar.

Sp. Tenuior.

Spong. Ust.

Stannum. Pp.

Styrax. Res.

Succin. Ppt.

Sulph. Lotum.

Sulp. Præc.

Sulph. Rotun.

Sulph. Sublim.

Sulph. Vivum.

Syrupus.

Syr. Althæ.

Syr. Caryoph.

Syr. Cort. A.

Syr. Croci.
Syr. Papav.
Syr. Limon. S.
Syr. Mori.
Syr. Rhamni.
Syr. Rhæad.
Syr. Rosæ.
Syr. Rhæi.
Syr. Rhei.
Syr. Rutæ.
Syr. Sambuc.
Syr. Sennæ.
Syr. Simplex.
Syr. Tolut.
Syr. Violæ.
Syr. Zingib.
Tapioca.
Terebin. Can.
Tereb. Chia.
Tereb. Vul.
Tereb. Venet.
Test. Ost. Pp.
Test. Præp.
Tr. Aloes.
Tr. Aloes. C.
Tr. Aurant.
Tr. Asæfœtid.
Tr. Bals. Peru.
Tr. Bals. Tolu.
Tr. Benzoes. C.
Tr. Calumb.
Tr. Camph. C.
Tr. Capsici.
Tr. Cardam.
Tr. Cardam. C.
Tr. Cascaril.
Tr. Castor.
Tr. Catechu.
Tr. Cinchon.
Tr. Cinchon. C.
Tr. Cinnam.
Tr. Cinnam. C.
Tr. Croci.
Tr. Digital.
Tr. Ferr. Amm.
Tr. Ferr. Mur.
Tr. Fuligin.
Tr. Galban.
Tr. Gentian. C.
Tr. Guaiac.
Tr. Guaiac. Am.
Tr. Helleb. N.
Tr. Humuli.
Tr. Hyoscy.
Tr. Jalapi.
Tr. Kino.
Tr. Lyttæ.
Tr. Myrrh.
Tr. Myrrh. c Al.
Tr. Opii.
Tr. Quassiæ.

Tr. Rhei.

Tr. Sabinæ. C.

Tr. Scillæ.

Tr. Sennæ.

Tr. Sennæ C.

Tr. Serp. Virg.

Tr. Valer. Amm.

Tr. Valerian.

Tr. Zingib.

Troch. Amyl.

Troch. Cretæ.

Troch. Glycyr.

Troch. Glyc c Opio.

Troch. Ipecac.

Troch. Lavand.

Troch. Mag.

Troch. Nitr.

Troch. Pontef.

Troch. Pateros.

Troch. Paregor.

Troch. Rosæ.

Troch. Sulph.

Troch. Tamar.

Troch. Tolut.

Troch. Zingib.

Tutiæ. Ppt.

Vegetab.

Vin. Aloes.

Vin. Aloet. Alk.

Vin. Antim.

Vin. Ferri.

Vin. Ipecac.

Vin. Opii.

Vin. Veratri.

Vinum. Rhei.

Uvæ. Passæ.

Ung. ex Althæ.

Ung. Camphor.

Ung. Cetac.

Ung. Ceræ.

Ung. Elemi. C.

Ung. Flor. Samb.

Ung. Hyd. Nit. Ox.

Ung. Hyd. F.

Ung. Hyd. Pr. A.

Ung. Hyd. M.

Ung. Lyttæ.

Ung. Pic. Liq.

Ung. Pic. Arid.

Ung. Resin. N.

Ung. Sambuc. V.

Ung. Sulph.

Ung. Sulph. C.

Ung. Tutiæ.

Ung. Veratri.

Ung. Zinci.

Zincum.

Zinc. Oxyd

Zinc. Sulph.

P. Rhabarb.

P. Sal. Nitri.

P. Sang. Drac.

P. Scam. c. Cal.

P. Scam. C. Aloes.

P. Scammon. C.

P. Salep.

P. Sem. Staph.

P. Sennæ. C.

P. Sem. Lini.

P. Sem. Anisi.

P. Sem. Cymen.

P. Sperm. Cæti.

P. Stanni.

P. Succin. C.

P. Tragac. C.

P. Uva. Ursi.

P. Valerian S.

P. Zingib.

P. Quassia.

Pip. Alb.

Pip. Long.

Pip. Nigrum.

Pip. Cayan.

Pix. Burgund.

Pixides.

Pimento.

Phials.

R. Althæa.

R. Ang. Hysp.

R Anchusa.

R. Bistor.

R. Caryoph.

R Cal. Arom.

R. Contray.

R. China.

R. Colomb.

R Curcum.

R. Gentian.

R. Ginseng.

R. Enulæ. C.

R Eryngium.

R. Galang.

R. Glycyrrh.

R Helleb. N.

R Helleb. A.

R Ipecac.

R. Jalappi.

R. Irid. Flor.

R. Mezer.

R. Pyreth.

R. Rhubarb.

R. Rubia. Tr.

R. Scillæ.

R Serpent. V.

R. Sars. Inc.

R. Senekæ.

R. Torment.

R. Valer. S.

R Zedoar.

R. Zingib.

R. Salep.

Ras. Corn. C.

Ras. Sant. Rub.

Ras. Guaiac.

Rass. Sassaf.

Resin. Jalep.

Resinæ. Fl.

Sp. Æth. Vit.

Sp. Æth. V C.

Sp. Æth. Nit.

Sp. Ammon.

Sp. Ammon. C.

Sp. Amm. Fœt.

Sp. Amm. Suc.

Sp. Anisi. C.

Sp. Camphor.

Sp. Carui.

Sp. Cinnam.

Sp. Cochlear.

Sp. Junip. C.

Sp. Lavend. C.

Sp. Lavend.

Sp. Menth. Pip.

Sp. Menth. Sat.

Sp. Nuc. Mosc.

Sp. Pimento.

Sp. Pulegii.

Sp. Raphan. C.

Sp. Rosimar.

Sp. Vin. Rec.

Sp. Vin. Ten.

Sago.

Sang. Dracon.

Sperm. Cæt.

Sem. Anethi.

Sem. Anisi.

Sem. Card. Maj.

Sem. Card. Min.

Sem. Carui.

Sem. Coriand.

Sem. Cydonior.

Sem. Cymini.

Sem. Dauci.

Sem. Fænug.

Sem. Fœnic. D.

Sem. Lini.

Sem. Santon.

Sem. Sinap.

Sem. Pœoniœ.

Sem. Staph.

Sal. Absinth.

Sal. Acetos.

Sal. C. C. Vol.

Sal. Epsom.

Sal. Nitri.

Sal. Prunel.

Succ. Hispan.

Succin. Pp.

Spong. Ust.

Spongia.
Sapo. Nig.
Sapo. Castill.
Stannum. Pp.
Soda. Phosph.
Sulph. Ant. Pp.
Sulph. Vivum.
Sulph. Rotun.
Sulph. Præcæp.
Syr. Althæ.
Syr. Caryoph.
Syr. Cort. A.
Syr. Croci.
Syr. Limon.
Syr. Mori.
Syr. Papav. E. R.
Syr. Papav. Alb.
Syr. Rosæ.
Syr. Rhæi.
Syr. Rutæ.
Syr. Simplex.
Syr. Spin. C.
Syr. Tolut.
Syr. Violæ.
Syr. Zingib.
Syr. Sambuc.
Tr. Aloes.
Tr. Aloes. C.
Tr. Assafœtid.
Tr. Cort. Aur.
Tr. Bals. Peru.
Tr. Bals. Tolu.
Tr. Benzoes. C.
Tr. Canthar.
Tr. Cardam.
Tr. Cardam. C.
Tr. Cascaril.
Tr. Castor.
Tr. Catechu.
Tr. Cinchon.
Tr. Cinchon. C.
Tr. Cinnam.
Tr. Cinnam. C.
Tr. Croci.
Tr. Colomb.
Tr. Digital.
Tr. Ferr. Amm.
Tr. Ferr. Mur.
Tr. Fuligin.
Tr. Galban.
Tr. Gentian. C.
Tr. Guaiac. Am.
Tr. Helleb. N.
Tr. Kino.
Tr. Jalipii.
Tr. Lavend. C.
Tr. Myrrh.
Tr. Myrrh. c. Al.
Tr. Opii.

Tr. Opii. Camp.

Tr. Rhabarb.

Tr. Rhabarb. C.

Tr. Sabinæ. C.

Tr. Sennæ. C.

Qr. Styptic.

Tr. Scillæ.

Tr. Sennæ.

Tr. Serp. Virg.

Tr. Valer. Amm.

Tr. Valerian.

Tr. Zingib.

Test. Ost. Pp.

Tutiæ. Ppt.

Tapioca.

Tereb. Chia.

Tereb. Vul.

Tereb. Venet.

Troch. Amyl.

Troch. Glycyrr.

Troch. Cretæ.

Troch. Mag.

Troch. Nitr.

Troch. Sulph.

Vin. Antim.

Vin. Ant. Tart.

Vin. Aloes.

Vin. Ferri.

Vin. Aloet. alk.

Vin. Rhabarb.

Vin. Ipecac.

Vit. Alb.

Vit. Cœrul.

Vit. Viridiæ.

Varæ.

Ung. Adip. Suilæ.

Ung. ex. Althæ.

Ung. Sabinæ.

Ung. Camphor.

Ung. Calc. Hyd. A.

Ung. Canthar.

Ung. Ceræ.

Ung. Cerus. Ac.

Ung. Elemi. C.

Ung. Flor. Samb.

Ung. Helleb. A.

Ung. Hyd. F.

Ung. Hyd. M.

Ung. Hyd. N.

Ung. Picis.

Ung. Resinæ. F.

Ung. Resinæ. N.

Ung. Sambuc. V.

Ung. Sperm. Cet.

Ung. Sulph.

Ung. Tutiæ.

Zinc. Calc.

Zinc. Vit. P.

Zinc. Vit.

### ORNAMENTED SHOP LABELS

FOR

### *DRAWERS AND BOTTLES;*

SELECTED FROM THE LAST EDITION OF THE

### *Edinburgh Pharmacopœia.*

ADAPTED TO THE

SHOPS OF SURGEONS, APOTHECARIES, AND DRUGGISTS,

*Of the same Pattern and Colour with those of the London.*

AC. SULPHUR.

Ac. Sulph. Ar.

Ac. Sulph. Dil.

Æsc. Hippocas.

Æther. Sulph.

Æth. Sulph. c. Alc.

Æth. Sul. c. Al. Ar.

Alcohol.

Alcoh. Ammon.

Alc. Amm. Arom.

Alc. Amm. Fæt.

Alcoh. Dilut.

Aloe. Hepat.

Aloe. Socot.

Alum. Ust.

Acet. Hydrar.

Acet. Plumb.

Acet. Potas.

Acet. Arom.

Acet. Scill. M.

Acid. Acetos.

Ac. Acet. Camph.

Ac. Acet. Dest.

Ac. Acet. Fort.

Ac. Benzoic.

Ac. Muriat.

Ac. Nitric.

Ac. Nitros.

Ac. Nitr. Dil.

Ac. Succini.

Ammonia. PP.

Ammon. Cupri.

Amygd. Com.

Amyr. Gilead.

Amylum.

Antimon. PP.

Aq. Acet. Amm.

Aq. Ammon.

Aq. Calcis.

Aq. Carb. Amm.

Aq. Cit. Auran.

Aq. Cit. Medic.

Aq. Destill.

Aq. Laur. Cass.

Aq. Laur. Cinnam.

Aq. Menth. Pip.

Aq. Men. Puleg.

Aq. My. Piment.

Aq. Potassæ.

Aq. Rosæ. Cen.

Aq. Sup. Car. Pot.

Aq. Sup. Car. Sod.

Arnica Mont.

Art. Absinth.

Art. Santon.

Bacc. Junip. C.

Bacc. Lauri. N.

Bals. My. Peru.

Bals. Sty. Benz.

Bals. Styr. Of.

Bit. Petrol.

Bolet. Igni.

Boras Sodæ.

Cancr. Lap. PP.

Capsic. Ann.

Carb. Ligni.

Carb. Ammon.

Carb. Barytæ.

Carb. Calc. PP.

Carb. Ferri.

Carb. Fer. Præc.

Carb. Ferr. PP.

Carb. Magnes.

Carb. Potas.

Car. Potas. Im.

Car. Potas. Pur.

Carb. Sodæ.

Carb. Zinc. Im.

Car. Zin. Im. PP.

Castor. Fib.

Centaur. Ben.

Cera. Alba.

Cera. Flava.

Cer. Car. Zin. Im.

Cerat. Simpl.

Coccus. Cacti.

Cons. Cit. Aurr

Cons. Rosæ C.

Cons. Rosæ G.

Corn. Cerv. El.

C. Angustur.

C. Canel. Alb.

C. Cinchon. Car.

C. Cinchon C.

C. Cinchon. Fl.

C. Cinchon. Rub.

C. Cit. Araunt.

C. Cit. Medic.

C. Crot. Eleuth.

C. R. Dap. Mezier.

C. Geoffr. In.

C. Laur. Cass.

C. Laur. Cinnam.

C. Pun. Granat.

C. Quas. Simar.

C. Querc. Rob.

C. Sambuc. Nig.

C. Swiet. Feb.

C. Swiet. Mahag.

C. In. Ulmi. Cam.

C. Winter. Aro.

Cuc. Colocyn.

Dat. Stramon.

Dec. Cinch. Of.

Dolichos. Pr.

El. Aromat.

El. Cass. Fist.

El. Cass. Senn.

El. Mim. Catec.

El. Opiat.

Emp. Assæf.

Emp. Gummos.

Emp. Hydrarg.

Emp. Meloe. V.

Emp. Meloe. V. C.

Emp. Ox. Ferr. R

Emp. Ox. Plumd S.

Emp. Resinos.

Emp. Saponac.

Emp. Simplex.

Emul. Amygd. C.

Emul. Camphor.

Emul. Mimos. Ni.

Ex. Anthem. Nob.

Ex. Cas. Sennæ.

Ez. Cinchon. Of.

Ex. Conv. Jalap.

Ex. Gentian. L.

Ex. Glycyr. Gl.

Ex. Hæmat. Cam.

Ex. Helleb. Nig.

Ex. Papav. Somn.

Ex. Rutæ. Grav.

Fl. Anthem. Nob.

Fl. Arnic. Mon.

Fl. Di. Caryoph.

Fl. Lavand. Sp.

Fl. Pun. Granat.

Fl. Sambuc. Nig.

Fl. Viol. Odor.

Fol. Aconit. N.

Fol. Althæ. Of.

Fol. Arb. Uv. Urs.

Fol. Asar. Eur.

Fol. At. Bellad.

Fol. Cardam. Pr.

Fol. Cas. Senn.

Fol. Conii. Mac.

Fol. Digit. Pur.

Fol. Jun. Sabin.

Fol. Lactuc. V.

Fol. Laur. Nob.

Fol. Malv. Syl.

Fol. Mellis. Of.

Fol. Menth. Pip.

Fol. Menyan. Tr.

Fol. Nicot. Tab.

Fol. Rhod. Chyr.

Fol. Rhi. Toxic.

Fol. Salv. Of.

Fol. Tanacet. V.

Fol. Tussil. F.

Fruc. Cuc. Col.

Fruc. Fic. Car.

Fruc. Prun. Dom.

Gallæ.

Gambogia.

Gratiola.

G. Ammoniac.

G. Ast. Tragac.

G. Bub. Galban.

G. Con. Scammon.

G. Fer. Assaf.

G. Junip. Lyc.

G. Mimos. Nil.

G. Myrrhæ.

G. Sagapeni.

Hordeum. Dis.

Hydrarg. Pur.

Hyoscyam. Nig.

Hyssop. Of.

Hydros. Ammon.

Inf. Cinchon. O.

Inf. Gentian. C.

Inf. Rhei. Pal.

Inf. Rosæ. Gal.

Inf. Tamar. c. S.

Kino.

Laur. Camphor.

Lign. Guaiac. O.

Lign. Hæmat. Cam.

Lign. Laur. Sass.

Lign. Quass. Exc.

Lign. Pt. Sant.

Lign. La Sass.

Lim. Ferri. Pur.

Lim. Stanni.

Lin. Aq. Calcis.

Lin. Simplex.

Magnesia.

Malva. Sylv.

Manna.

Marrub. Vulg.

Meloe. Vesic.

Momor. Elater.

Moschus. Mos.

Mim. Catechu.

Muc. Mimos. Nil.

Mur. Ammon.

Mur. Amm. et Fer.

Mur. Antimon.

Mur. Barytæ.

Mur. Hydrarg.

Mur. Sodæ.

Myrt. Piment.

Nitr. Argent.

Nitr. Potas.

Nux. Myrist. Mos.

Ol. Ammoniat.

Ol. Amygd. C.

Ol. Camphor.

Ol. Coci. Butyr.

Ol. Lini. Usit.

Ol. Lauri. Nob.

Ol. Olæ. Europ.

Ol. Ricini. C.

Ol. Succini.

Ol. Succin. Pur.

Ol. Sulphur.

Ol. Terebinth.

Ol. Vol. Cit. Med.

Ol. V. Caryoph. A.

Ol. V. Junip. C.

Ol. V. Jun. Sabin.

Ol. V. Lavend. Sp.

Ol. V. Lau. Sass.

Ol. V. Mel. Leuc.

Ol. V. Menth. Pip.

Ol. V. Myr. Mos.

Ol. V. Myr. Pim.

Ol. V. Pim. Anisi.

Ol. V. Pini. Pur.

Ol. V. Rorism. Of.

Opium.

Orig. Majoran.

Ovis. Ar. Adeps.

Ox. Ant. c. Ph. Cal.

Ox. Ant. c. Sul. P. N. P.

Ox. Ant. c. Sul. Vit.

Ox. Ant. Vit. c. Cer.

Ox. Arsenici.

Ox. Ferr. Nig. Pur.

Ox. Ferr. Nigg.

Ox. Hydrar. Cin.

Ox. Hyd. R. P. Ac. Ni.

Ox. Plumb. Alb.

Ox. Plumb. Rub.

Ox. Plumb. Semiv.

Ox. Zinci.

Ox. Zinc. Imp.

Ox. Zinc. Imp. P. P.

Papav. Somnif.

Phosph. Sodæ.

P. Aloes. et Asaf.

P. Aloes. et Myr.

P. Aloetic.

P. Amm. Cupri.

P. Assafœt. C.

P. Hydrarg.

P. Opiat.

P. Rhei. C.

P. Scillit.

Piper. Long.

Piper. Nigr.

Pix. Liquida.

Potassa.

Pot. c. Calce.

Pot. Carb. Calc.

P. Acor. Calam.

P. Amom. Zingib.

P. Amygdal C.

P. Angustur.

P. Anth. Pyreth.

P. Antimon.

P. Aromat.

P. Aris. Serpent.

P. Asari. C.

P. Canel. Alb.

P. Carb. Calc. C.

P. Caryoph. Ar.

P. Cass. Sennæ.

P. Castor.

P. Cinchon. Of.

P. Cinchon. F.

P. Cinchorn. R.

P. Colombæ.

P. Conii. Mac.

P. Con. Jalapæ.

P. Crot. Eleuth.

P. Del. Staphis.

P. Digit. Purp.

P. Dor. Contraj.

P. Gallarum.

P. Gentian, L.

P. Glycyrrh. Gl.

P. G. Guaiac. Off.

P. Helleb. Nig.

P. Jalapæ. C.

P. Ipecacuan.

P. Ipec. et Opii.

P. Junip. Sabin.

P. Laur. Cinnam.

P. Lini. Usit.

P. Maloes. Ves.

P. G. Mimos. Nil.

P. G. Myrrhæ.

P. Opii.

P. Opiatus.

P. Rhei. Palm.

P. Scammon. C.

P. Scillæ. Mar.

P. Quass. Simar.

P. Sinap. Alb.

P. Stanni.

P. Sulph. Al. C.

P. Torment. Er.

P. Valerian. Of.

P. Veratr. Alb.

P. Zingib.

R. Acor. Cal.

R. Allii. Sat.

R. Althæ. Off.

R. Amom. Zing

R. Anch. Tinct.

R. Ang. Arcang.

R. Ant. Pyreth.

R. Ap. Petros.

R. Arct. Lapp.

R. Ar. Serpent.

R. Cochl. Arm.

R. Colch. Aut.

R. Colombæ.

R. Con. Jalap.

R. Dor. Contraj.

R. Gentian. L.

R. Glycyr. Gl.

R. Helleb. N.

R. Ipecacuan.

R. Irid. Flor.

R. Lyon. Tarax.

R. Lobel. Syph.

R. Pol. Seneg.

R. Pol. Bistor.

R. Pol. Filix. M.

R. Rhei. Palm.

R. Rub. Tinct.

R. Scillæ. Mar.

R. Sm. Sarsap.

R. Spigel. Mar.

R. Torment. Er.

R. Valer. Off.

R. Verat. Alb.

Res. Guaiac. Of.

Res. Pini.

Res. Pier. Draco.

Res. L. Pin. Ab.

Res. L. Pin. Bals.

Res. L. Pin. Lar.

Rosa. Centif.

Rosa. Gallic.

Rosmarin. Off.

Rub. Ferri. Pp.

Sacch. Non. Pur.

Sacch. Puriss.

Sapo.

Scill. Mar. Ex.

Sem. Amom. Rep.

Sem. An. Fœnic.

Sem. Oven. Sot.

Sem. Car. Carui.

Sem. Coriand. Sat.

Sem. Dau. Carot.

Sem. Del. Staph.

Sem. Lini. Usit.

Sem. Sinap. Alb.

Sol. Acet. Zinc.

Sol. Mur. Baryt.

Sol. Mur. Calcis.

Sol. Sulp. Cupr.

Sol. Sulp. Zin.

Sperma. Ceti.

Sp. Æther. Nit.

Sp. ar. Carui.

Sp. Junip. C.

Sp. Laur. Cinnam.

Sp. Lavand. Sp.

Sp. Lavand. C.

Sp. Menth. Pip.

Sp. Myrist. Mos.

Sp. Myrt. Piment.

Sp. Rorism. Off.

Spongia. Off.

Squam. Fer. P.

Subac. Cupri.

Submur. Hydr.

Subm. Hyd. Præc.

Sub. Sul. Hyd. F.

Succinum.

S. Cochlear. C.

S. S. Acon. Nap.

S. S. Atr. Bell.

S. S. Conh. Mac.

S. S. Hyos. Tigr.

S. S. Lact. Vir.

S. S. M. Elater.

S. S. Samb. Nigr.

Suis. Scrof. Adeps.

Sulph. Alum.

Sulph. Alum. Ex.

Sulph. Baryt.

Sulph. Cupri.

Sulph. Ferri.

Sulph. Ferri. Ex.

Sulph. Magnes.

Sulph. Potass.

Sul. Pot. c. Sulph.

Sulph. Sodæ.

Sulph. Zinci.

Sulphur. Subl.

Sulp. Subl. Lot.

Sulph. Antim.

Sulph. Ant. Præc.

Sulph. Antim. PP.

Sulph. Hydr. N.

Sulph. Hydr. R.

Sulph. Potass.

Sum. Ch. Cent.

Sum. Spart. Scop.

Sup. Tar. Pot. Im.

Sup. Tar. Potass.

Syr. Acid. Acet.

Syr. Althæ. Of.

Syr. Amom. Zing.

Syr. Cit. Aurant.

Syr. Cit. Med.

Syr. Colch. Aut.

Syr. Di. Caryop.

Syr. Papav. Som.

Syr. Rhamn. Cat.

Syr. Rosæ. Cent.

Syr. Rosæ. Gal.

Syr. Scil. Mar.

Syr. Simplex.

Syr. Tolu. Bals.

Syr. Violæ. Od.

Tamarin. Ind.

Tartr. Antim.

Tartr. Potass.

Tartr. Pot. et Sod.

Tr. Aloes. Æther.

Tr. Aloes. et Mur.

Tr. Aloes. Soc.

Tr. Amon. Rep.

Tr. Ar. Serpent.

Tr. Benzoin. C.

Tr. Camphoræ.

Tr. Castorei.

Tr. Castor. C.

Tr. Cinchon. Of.

Tr. Cinnam. C.

Tr. Colombæ.

Tr. Conv Jalap.

Tr. Croc. Angl.

Tr. Digit. Purp.

Tr. Fer. Assaf.

Tr. Gentian. C.

Tr. Guaiac. Of.

Tr. Guaiac. Am.

Tr. Helleb. Nigr.

Tr. Hyoscy. Nigr.

Tr. Kino.

Tr. Laur. Cinnam.

Tr. Meloes. V.

Tr. Mim. Catechu.

Tr. Mur. Ferri.

Tr. Myrrhæ.

Tr. Opii.

Tr. Opii. Ammon.

Tr. Rhei et Al.

Tr. Rhei et Gent.

Tr. Rhei. Palm.

Tr. Saponis.

Tr. Sapon et Opii.

Tr. Sennæ. C.

Tr. Toluif. Bals.

Tr. Veratri. Alb.

Troch. Carb. Calc.

Troch. Glycyr. Gl.

Troch. Glycyr. c. Op.

Troch. Gummos.

Troch. Nitr. Pot.

Ung. Acet. Nit.

Ung. Acet. Plumb.

Ung. Hydrarg.

Ung. Inf. Melovi.

Ung. Nit. Hydr. F.

Ung. Nit. Hydr. M.

Ung. Ox. Hyd. Cin.

Ung. Ox. Hyd. Rub.

Ung. Ox. Plumb. Al.

Ung. Ox. Zinci.

Ung. Ox. Zinci. Im.

Ung. Picis.

Ung. P. Meloes.

Ung. Resinos.

Ung. Simplex.

Ung. Subac. Cup.

Ung. Sulphuris

Ung. Vara.

Vin. Aloes. Soc.

Vin. Gentian. C.

Vin. Ipecac.

Vin. Nic. Tabac.

Vin. Rhei. Palm.

Vin. Tartr. Ant.

Zincum.

May be had of the Publisher, St. Thomas's Street, Borough; or of Mr. ADAM BLACK, Edinburgh.

### WINDOW LABELS,

*ON GREEN ORANGE AND WHITE PAPER.*

CHELTENHAM SALTS

ESSENTIAL SALT OF LEMONS

FISH SAUCES

GENUINE PATENT MEDICINES

HUXHAM'S TINCTURE OF BARK

ICELAND MOSS

INDIAN ARROW ROOT

ISINGLASS

ICE CREAM

LAVENDER WATER

LOZENGES

LEECHES

MEDICINES FOR CATTLE

NEW HONEY

OILS AND PICKLES

PERFUMERY

PRESCRIPTIONS ACCURATELY PREPARED

PREPARED GINGER

REFINED LIQUORICE

SPRUCE BEER

SODA WATER

WIMDSOR SOAP

BLANKS

*See Specimen given with this Catalogue.*

### ENGLISH LABELS,

FOR

### SURGEONS, APOTHECARIES, CHEMISTS AND DRUGGISTS,

*Of such Sizes and Patterns as the respective Articles may require,*

AT PER HUNDRED.

AROMATIC VINEGAR.
——— Species.
Asthmatic Elixir.
Anodyne Liniment.
Arqubusade.
Æthiop's Minerals.
Æther.
Adhesive Plaister.
Aqua Fortis.
Aperint Moisture.
Best Bitters.
—— Honey.
—— Rose Leaves.
—— Æther.
—— Bark.
Butyer of Antimony.]
Blistering Cerate.
——— Plaister.
Balsam of Capaiva.
——— Gilead.
——— Riga.
——— Elixir.
Blank Labels of various patterns.
Castor Oil.
Cold Drawn Castor Oil.
Calcined Magnesia.
Corrossive Sublimate.
Camphor Julip.
Cream of Tartar.
Colomel.
Colomel Pills.
Cough Drops.
Collyreum.
Comp. Powder of Chalk.
Chrystallized Acid of Lemons
Conserve of Hips.
——— Roses.
Candid Horehound.
Drawing Ointment.
Diachylon and Gum.
——— Plaister.
Draught.
Elizir Vitriol.
———Acid.
—— Proprietus.
—— Aloes.
Extract of Saturn.
Extract of Lead.
——— Bark.
——— Roses.
Emetic Tartar.
——— Draught.
——— Wine.
Embrocation.
———for the Eyes.
Eau de Luce.
Elder Ointment.
Fryar's Balsam.
Fit Drops.
Flower Sulphur.
Flake Manna.
Fine Allum.
—— Virgin Honey.
Goulard.
——— Extract.
——— Cerate.
Gout Cordial.
Gold Beater's Skin.
Gum Plaister.
Ginger Seeds.
Gouland's Lotion.
Hoffman's Ætherial Anodyne
——— Æther.
——— Anodyne Liquor.
Honey of Roses.
Healing Ointment.
Hartshorn Water.
Indian Arrow Root.
Jesuit's Drops.
Infusion of Roses.
Knife and Spatula.
Laudanum.
Linitive Electuary.
Lint.
Liniment.
Laxative Draught.
——— Mixture.
Luna Caustic.
Lemon Juice.
Lime, ditto.
Lotion.
Lavender Drops.
Lozenges Magnesia.
——— Tolu.
——— Pectoral.
Lozenges Patirosa.
——— Rose.
——— Peppermint
——— Tamarind.
——— Bath.
——— Lavender.
——— Ginger.
——— Cardialgic.
——— Black Currant.
——— Nitre.
——— Anniseed.
——— Lemon.
——— Dawson's.
——— Coltsfoot.
——— Poppy.
——— Steel.
——— Spermaceti.
——— Cinnamon.
——— Ginger.
——— Clove.
——— Acidulated Rose.
Mortar and Pestle.
Manna.
Magnesia.
Mixture.
——— for the Grease.
Mercurial Ointment.
——— Pills.
Marshmallow Ointment.
Mineral Water.
Opodeldoc.
Oxymel Squills.
Opium Pills.
Oil of Almonds.
——— Jessamine.
——— Orange.
——— Thyme.
——— Roses.
——— Cassia.
——— Tuberose.
——— Peppermint.
——— Amber.
——— Cloves.
——— Turpentine.
——— Camphor.
——— Lavender.
——— Anniseed.
——— Tartar.

Oil of Carraway.
—— Vitriol.
Pectoral Lozenges of Tolu.
Powder Turkey Rhubarb.
—— Russia Rhubarb.
—— Antimonial.
—— Dover's.
—— Drying.
—— Jalap.
—— Yellow Bark
—— Indian Root.
Powder James's.
—— Ipecacuhana
—— Pale Bark.
—— Rhubarb.
—— Ginger.
—— Bark.
—— Arsenic.
—— Gascoign.
—— Aromatic.
—— for Worms.
—— Tin.
—— Cinnamon.
—— Myrrh.
—— Injection.
—— Gum Arabic.
—— Salt Petre.
—— Colombo.
—— of Stalk Root
Paregoric Elixir.
Pontifract Cakes.
Peppermint Drops.
—— Seeds.
—— Pipes.
—— Perls.
Pile Ointment.
Purging Draught.
—— Ball.
—— Pills.
Palsy Drops.
Preserved Ginger.
Prepared, ditto.
Purified Epsom Salts.
—— Cheltenham, ditto.
Red Precipitate.
Rufus's Pills.
Refined Liquorice.
Spirits Lavender Compound.
Spirits of Aromatic Vinegar for the Head-ach.
—— Amber.
—— Camphor.
—— Hartshorn.
Spirits of Lavender.
—— Mindererus
—— Sal Volatile.
—— Turpentine.
—— Wine.
—— Wine Rect.
—— Salts.

Spirits of Wine Camph.
—— Sal Ammoniac.
Steer's Opodeldoc.
Sugar of Lead.
Supercarbonate Kali.
—— Potass.
—— Soda.
Steel Comfits.
Scales and Weights.
Sundries.
Soap Plaister.
Salts Glauber.
—— Polychrist.
—— Rochell.
—— Smelling.
—— Essential of Lemons.
—— Tartar.
—— Wormwood.
—— Steel.
—— Tasteless Purging.
—— Epsom.
—— Cheltenham.
—— Volatile.
Stomatic Bitters.
—— Elixir.
Soluble Tartar.
Senna.
—— Leaves.
Spermaceti Ointment.
Syrup of Violets.
—— Tolu.
—— Saffron.
—— Poppies.
—— Balsam.
—— Buckthorn.
—— De Vinegre.
—— Orgeat.
—— Marshmallows.
—— Squils.
—— Rhubarb.
Tincture Aloes
—— Assafœtida
—— Bark
—— Benjamin C.
—— Cardamoms
—— Columbo
—— Guaiacum
—— —— Vol.
—— Gentian
—— Jalap
—— Myrrh
—— Opium
—— Rhubarb
—— Senna
—— Sacra
—— Steel
—— Balsam Tolu
—— Valerian Vol.
—— Valerian
—— for Wounds

Tincture Aromatic
—— Styptic
—— of Turkey Rhubarb
—— Quassia
—— Ginger
—— Myrrh, for the Teeth and Gums
—— Squills
—— Comp. Bark
—— of Bark, Whytt's
—— of Bark, Huxham
—— —— for Gout
—— Hops
Tincture of Steel, muriated
—— Hiera Picra
—— Cascanilla
—— Stomachicum
Turkey Rhubarb
Tooth Opiate
Turlington's Balsa
Tramatic ditto
The Drops
—— Lotion
Tapioca
Venice Treacle
Vitriolic Æther
Vol. Aromatic Spirits
—— Liniment
Water, Aniseed
—— Arquebusade
—— Cinnamon
—— Dill
—— Eye
—— Elder Flower
—— Hungary
—— Hysop
—— Hysteric
—— Lavender
—— Mint
—— Ita Orange Flow.
—— Nutmeg
—— Pennyroyal
—— Peppermint
—— Rose
—— Orange Flower
—— Lime
—— Honey
—— Best Cinnamon
Wax Ointment
White Precipitate
Wine, Antimonial
—— Aloetic
—— Steel
—— Indian Root
—— Rhubarb
Yellow Basilicon

*The above may be had of either Pattern in Copper Plate Title, marked*

A. B. C.

### LABELS FOR PERFUMERS.

Almond Paste
Best Double Distill'd Lavender Water, for Pint Bottles
Ditto, for Half Pints
Bears' Grease
Carmine
Cold Cream
Eau de Sauve
——— Arquebusade
Espirit de la Rosu
Extract of Roses
Elder Flower Water
Essence Lemon
——— Bergamot
——— Cypre
——— Tuberose
——— Jessamine
——— Musk
——— Lavender
——— Pennyroyal
——— Peppermint
——— Jonquil
——— Violets
——— Roses
——— Millefleur
——— Duchesse
——— Marechalle
——— Cloves
——— Orange
——— Helitrope
——— Vanille
——— Bouquet
——— Thyme
Essence Ambergrease
——— Cinnamon
Face Powder
Gold Beaters' Skin
Genuine Otto of Roses
Huile Antique a la Rose
Huile Antique au Millefleurs
Huile Antique a la Tuberose
Huile Antique a la fleur D'Orange
Huile Antique au Jasmin
Huile Antique a la Violette
Hungary Water
Lip Salve
Milk of Roses, coloured Paper
Prepared Charcoal
Pearl Powder
Pearl Dentifrice, coloured Paper
Plate Powder
Poudre de la Marshalle
Pomade Divine
Pomatums, Cowslip
——— Rose
——— Violet
——— Sweet-sc.
——— Lily
——— Bergamot
——— Marrow
——— Carnation
——— Lemon
——— French
——— Jessamine
Pomatum Citron
Rouge
Rouge Vegetable Liquid
Rose Water, for Pints
Ditto, for Half Pints
Soaps, Windsor
——— Violet
——— Naples
——— Rose
Superfine Plain Powder
——— French ditto
——— Violet ditto
——— Mareschalle ditto
——— Orris ditto
——— Rose ditto
——— Shaving Powder
Syrup de Capillaire
Tooth Powder, Violet
——— Rock
——— Rose
——— Wirtemberg
——— Prince Regent
——— Princess of Wales
——— Royal
——— Imperiel
——— Fine
——— Superfine
——— Asiatic
Rose Lip Salve
Princess of Wales's do.
Fine ditto

### OILS, PICKLES, &c.

Anchovies
Best Olive Oil
Best Eating ditto
Browning Sauce
Camp Vinegar
Capiicombs
Cauliflowers
Cavitch
Cavice
Cayan Pepper
Chillie Vinegar
Cucumbers
Cucumber Mangoes.
Coratch.
Distilled Vinegar
Elder Vinegar
Essence of Anchovies
Fine India Soy
Fine Lucca Oil
Fine Capers
Fine Spermaceti Oil
French Olives
French Beans
Four Thieves Vinegar
Garlic ditto
Gerkins
India Pickles
India Mangoes
Italian Fish Sauce
Ketchup
Lemon Pickle
Linseed Oil
Mixed Pickles
Melon Mangoes
Mushrooms
Mushroom Ketchup
Neatsfoot Oil
Onions
Oyster Ketchup
Piccadilly
Quince Sauce
Red Cabbage
Sallad Oil
Samphire
Sauce Piquant
Sauce Royal
Sauce Universal
Sauce Cheroker
Spanish Olives
Tarragon Vinegar
Truffle Sauce
Walnuts
Walnut Ketchup
White Wine Vinegar
Zoobditty Match

*Any of the above Lables may be had in four different Patterns.—One Specimen is given in Copper Plate Title, marked* D.

### CONFECTIONARY.

Apple Jelly
Apricot Jam
Black Currant Jelly
Cherry Bounce
Cold Cream
Currants
Carrant Jam
Damsons
Damson Cheese
Dried Cherries
Gooseberries
Gooseberry Jam
Green Sweetmeats
Guaiva Jelly
Orange Marmalet
Orgeat
Peach Jam
Pearl Apple
Plumb Jam
Preserved Cream
Preserved Ginger
Quince Jam
Raspberry Jam
Raspberry Jelly
Raspberry Vinegar
Red Currant Jelly
Robb
Strawberry Jam
West-India Sweet-meats

### SHOP LABELS FOR GROCERS AND OILMEN,

### ON GREEN, YELLOW, AND WHITE PAPER.

*In the same Pattern as the Latin.*

Allspice
Alum
Anniseed Lozenges
Bees Wax
Barley Sugar
Bath Lozenges
Bay Salt
Black Lead
Black Pepper
Bitter Almonds
Brown Candy
Brown Ginger
Bulls Eyes
Carolina Rice
Citron
Cloves
Corianders
Cardialgic Lozenges
Curry Powder
Cassia
Carraways
Cinnamon
Currants
Dawson's Lozenges
East India Rice
Emery
Faro Figs
Fine Mustard
French Barley
Ginger Lozenges
Ground Rice
Ground Ginger
Gum Arabic
Isinglass
Ivory Black
Jordan Almonds
Lamp Black
Lavender Lozenges
Lemon Peel
Long Pepper
Mace
Macaroni
Magnesia Lozenges
Malaga Raisins
Millet
Mint Lozenges
Mustard
Nitre Lozenges
Nutmegs
Orange Peel
Patirosa Lozenges
Pearl Barley
Pearl Ash
Peppermint Drops
Peppermint Lozenges
Poland Starch
Prunes
Powder Blue
Rose Lozenges
Salt Petre
Spanish Juice
S. Caraways
Shot
Stone Blue
Sun Raisins
Salt Prunella
Tamarind Lozenges
Tolu Lozenges
Turkey Figs
Vermicelli
Virgins Wax
White Candy
White Ginger
Yellow Oker

### *LIVERPOOL LINT,*

4s. 6d. per Pound.

*An Allowance of Twelve and a half per Cent. is made on Fourteen Pounds.*

THE FOLLOWING

### LABELS

ARE PRINCIPALLY DESIGNED FOR THE USE OF

### *WHOLESALE DRUGGISTS,*

At per Hundred.

Acet. Scillæ.

Acid. Acetic.

Acid. Muriat.

Acid. Nitros. F.

Acid. Nitric.

Acid. Sulph.

Æther. Sulph.

Antim. Mur.

Aq. Alexit.

Aq. Anethi.

Aq. Bryoni. C.

Aq. Carui.

Aq. Cinnam.

Aq. Fœniculi.

Aq. Flor. Aur.

Aq. Sambuc.

Aq. Mellis.

Aq. Menth. Pip.

Aq. Menth. Vir.

Aq. Pimentæ.

Aq. Pulegii.

Aq. Rosæ.

Aq. Lavand. Od.

Arqubusade.

Acid. Citric.

Acid. Oxalic.

Acid. Tartar.

Ammon. Carb.

Ant. Sulph. Præc.

Antim. Tartar.

Argent. Nit.

Ess. Limonis.

Ess. Bergam.

Ferri. Sulph.

Bals. Copaiba.

Bals. Canada.

Bals. Sulph.

Bals. Peru.

Bals. Tolu.

Ceratum.

Cerat. Calam.

Cerat. Cetac.

Cerat. Lyttæ.

Cerat. Plumb. S. A.

Cerat Plumb. Comp.

Cerat. Plumb. Comp.

Cerat. Resinæ

Cerat. Sabinæ.

Cerat. Saponis.

Cinnab. Ant. P. P.

Con. Aurant.

Conf. Amygd.

Conf. Cassie.

Conf. Sennæ.

Conf. Opii.

Cunf. Japon.

Conf. Rosæ. Can.

Conf. Rosæ. Gal.

Conf. Rutœ.

Conf. Scammon.

Cup. Ammon.

Eau. de. Luce.

Ex. Aconiti.

Ex. Aloes.

Ex. Anthem.

Ex. Belladon.

Ex. Cacum. G.

Ex. Cinchon.

Ex. Colocynth.

Ex. Colocynth. C.

Ex. Conii.

Ex. Elaterii.

Ex. Hæmato.

Ex. Humuli.

Ex. Rhei.

Ex. Sarsap.

Ex. Taraxaci.

Ferrum. Ammon.

G. Galban. Col.

Hydrargyrus.

Hyd. Nit. Oxyd.

Hyd. Oxyd. Cin.

Hyd. Oxyd. Rub.

Hyd. Submur.

Hyd. Vit. Flav.

Lin. Terebinth.

Lin. Camphor. C.

Lin. Sapon C.

Liq. Ammoniæ.

Liq. Antim. Tart.

Liq. Calcis.

Liq. Plumbi. Acet.

Liq. Potassæ.

Liq. Potassæ. Sub.

Liq. Vol. C. C. Opt.

Magnes. Calc.

Mel. Opt.

Mel. Rosæ.

Moschus. Chin.

Ol. Amygd.

Ol. Anisi.

Ol. Carui.

Ol. Caryoph.

Ol. Juniper.

Ol. Lavand. Ang.

Ol. Lavand. Ex.

Ol. Lini. S. I.

Ol. Anethi.

Ol. Carui.

Ol. Cajuputi.

Ol. Casslæ.

Ol. Pimentæ.

Ol. Menth. Pip.

Ol. Origani.

Ol. Olivæ. Op.

Ol. Olivæ. Sec.

Ol. Rosmarini.

Ol. Ricini. E. I. Opt.

Ol. Ricini. W. I. Opt.

Ol. Succini. R.

Ol. Terebinth.

Ol. Viridæ.

Ol. Pulegii.

Ol. Palmœ.

Ol. Rosmarini.

Ol. Succini. R.

Oxymel.

Oxymel. Scillæ.

P. Colocynth.

P. Cinchon. C.

P. Cinchon. L.

P. G. Acaciæ.

P. G. Myrrhæ.

P. Cinnam.

P. Cinnam. C.

P. Ipecac.

P. Ipecac. C.

P. Tragac. C.

P. Rhei. E. I. Opt.

P. Scillæ.

P. Zingib. Jam.

Pil. Aloes. Co.

Pil. Aloes c Myrrh.

Pil. Aloes c Coloc.

Pil. Camboglæ. C.

Pil. Ferri c Myrrh.

Pil. Galban. C.

Pil. Hydrarg.

Pil. Hyd. Submur.

Pil. Sapon. c Opii.

Pil. Scillæ. C.

Pil. Styracis.

Potassæ. Acet.

Pottassæ. Carb.

Potassæ. Subcarb.

Potassæ. Sulph.

Potassæ. Tart.

Potassa. Fusa.

Pulv. Opii.

Soda. Subcarb. Ex.

Sodæ. Carbon.

Sodæ. Subcarb.

Sp. Ammon. Arom.

Sp. Ammon. Fœt.

Sp. Anisi.

Sp. Armorac. C.

Sp. Camphoræ.

Sp. Carui.

Sp. Cinnam.

Sp. Juniperi. C.

Sp. Lavand.

Sp. Lavand. C.

Sp. Cochlear.

Sp. Menth. Pip.

Sp. Menth. Vir.

Sp. Myrist.

Sp. Pimentæ.

Sp. Pulegii.

Sp. Rosmarini.

Sp. Æther. Ar.

Sp. Æther. C.

Sp Æther. Nit.

Sp. Æther. S.

Sp. Vin. Rect.

Spongia. Usta.

Syr. Althææ.

Syr. Aurantii.

Syr. Croci.

Syr. Limonis.

Syr. Mori.

Syr. Marubii.

Syr. Papav.

Syr. Rhœados.

Syr. Rhamni.

Syr. Rhei.

Syr. Rosæ.

Syr. Sennæ.

Syr. Scillæ.

Syr. Tolut.

Syr. Violæ.

Syr. Zingib.

Syrupus.

Tamarind. Opt.

Terib c Chio.

Tereb. Venet.

Ung. Cetacei.

Tr. Aloes.

Tr. Aloes. C.

Tr. Assafætidæ.

Tr. Aurant.

Tr. August.

Tr. Benzoin. C.

Tr. Calumbæ.

Tr. Capsici.

Tr. Camphor.

Tr. Cardam. C.

Tr. Cascarillæ.

Tr. Castor.

Tr. Ferri. Amm.

Tr. Helleb. Nig.

Tr. Bals Tolut.

Tr. Digitalis.

Tr. Humuli.

Tr. Hyoscyami.

Tr. Jalapæ.

Tr. Lyttæ.

Tr. Myrrhæ. C.

Tr. Rhei. C.

Tr. Serpent.

Ung. Elemi. C.

Ung. Hydrarg.

Ung. Hyd. Mit.

Ung. Picis. Liq.

Ung. Sambuci.

Ung. Sulphur. C.

Vin Aloes.

Vin. Antim.

Vin. Ferri.

Vin. Ipecac.

Vin. Opii.

*The above Labels are of the Patterns* E. F. *in Copper Plate Title; besides which any other Names of the former, or present Pharmacopœia may be had to correspond.*

### *DIRECTIONS FOR PATENT MEDICINES, &c.*

Daffy's Elixir at per quire or ream
Godfrey's Cordial, ditto
———— Tops, ditto
Bateman's Drops, ditto
Turlingtons' Balsam, ditto
Steers's Opodeldoc, ditto
Stoughton's Elixir, ditto
British Oil, ditto
Freeman's Bathing Spirits, ditto
Huxham's Tincture of Bark, ditto
Dutch Drops
Essential Salt of Lemons
Dalby's Carminative, ditto
Scotch Pills, ditto
Essence of Peppermint, ditto
Fryer's Balsam, ditto
Hooper's Pills, ditto
Vegetable Acid, ditto
Essence of Anchovies, at per hundred
Iceland Moss, ditto
Indian Arrow Root, ditto
Essence of Spruce, ditto
Permanent Ink, ditto
Stramonium, ditto
Boot Top Liquid, ditto
Pink Dye, ditto
Liquid Blue, ditto
Nankeen Dye, ditto
Prepared Jamaica Ginger, ditto
Furniture Gloss, ditto

Black Lines for Labels, at per quire
Directions for Family Medicine Chests
Ditto for Sea Chests
Catalogue of Drugs, Latin
Ditto ditto, Latin and English, old and new Names
Druggists' Pocket Guide, containing a List of Duties, Drawbacks, &c. on Drugs
Coloured Paper, Green, Pink, Orange, Yellow, and Purple, in quires
Ditto, in Rolls
Tooth Powder Boxes, labelled and varnished, at per gross
Ditto ditto, No. 1.
Ditto ditto, No. 2.
Ditto Lip Salves ditto, No. 3.
Pill Boxes, four in a nest, at per ditto
Ditto, two in a nest, ditto
Ornamented Shop Labels in sets, as specified in a foregoing page
Labels for Horse Medicines

### *Medicine Stamps at the following Discount:*

10 per Cent. on £10.——7½ per Cent. on £5.——5 per Cent. on £1.

*Sent to any part of the Kingdom.*

ENGRAVING AND PRINTING EXECUTED IN THE NEATEST STYLE.

*MEDICAL BOOKS AT TEN PER CENT. DISCOUNT.*

